# Strengthening partnerships between substance use researchers and policy makers to take advantage of a window of opportunity

**DOI:** 10.1186/s13011-019-0199-0

**Published:** 2019-03-04

**Authors:** Zachary F. Meisel, Julia Mitchell, Daniel Polsky, Nada Boualam, Ellen McGeoch, Janet Weiner, Matthew Miclette, Jonathan Purtle, Bruce Schackman, Carolyn C. Cannuscio

**Affiliations:** 1 Center for Emergency Care Policy and Research, Perelman School of Medicine, University of Pennsylvania, Blockley Hall, 423 Guardian Drive, Room 413, 3400 Civic Center Blvd, Philadelphia, PA 19104-4865 USA; 2 Center for Health Economics of Treatment Interventions for Substance Use Disorder, HCV and HIV, Philadelphia, USA; 3 Leonard Davis Institute of Health Economics, 3641 Locust Walk, Philadelphia, PA 19104 USA; 4 Dornsife School of Public Health, Drexel University, 3215 Market St, Philadelphia, PA USA; 5 Weill Cornell Medical College, 425 East 61st Street, Suite 301, New York, NY 10065 USA; 6 Department of Family Medicine and Community Health, Perelman School of Medicine, University of Pennsylvania, 3620 Hamilton Walk, Philadelphia, PA 19104 USA

**Keywords:** Substance use disorder, Policy, Knowledge transfer

## Abstract

**Background:**

The National Institute on Drug Abuse has identified a persistent research-to-practice gap in the implementation of evidence-based prevention and treatment programs for substance use disorder. To identify mechanisms to close this gap, we sought to obtain and characterize the range of policy makers’ perspectives on the use of research in substance use disorder treatment and coverage decisions.

**Methods:**

We conducted open-ended, semi-structured interviews with a purposive sample of eighteen policy makers involved in the delivery of health services. The aim was to identify barriers and facilitators, attitudes, beliefs, and experiences surrounding the use of research related to the treatment and economics of substance use disorder.

**Results:**

The analysis generated four themes: 1) policy maker engagement with evidence and researchers; 2) strategic use and usefulness of research; 3) scientific rigor versus relevance; and 4) communication of evidence. Within each theme, the participants identified barriers, facilitators, current practice, and gave their perspectives on “ideal conditions” for research design, conduct and communication.

**Conclusions:**

Recommendations for investigators are the following actionable steps: 1) partner with policy makers early in the research process, 2) formulate and use research designs to meet the strategic goals of end-users; 3) systematically test alternative phrasing of scientific terminology – particularly in the realm of cost effectiveness research – that allow end users to better understand and repurpose the data; 4) incorporate qualitative research methods to uncover the narratives that explain the context and relevance of evidence; 5) incorporate study designs that prioritize timeliness of results; and 6) promote and reward researcher involvement in policy discussions.

**Electronic supplementary material:**

The online version of this article (10.1186/s13011-019-0199-0) contains supplementary material, which is available to authorized users.

## Background

Despite the growing sense of public alarm about the escalation of opioid use and overdose, evidence-based treatments remain underutilized [1–6]. A failure to apply the best evidence pervades substance use disorder (SUD) policy-making, financing, and delivery [6, 7]. The National Institute on Drug Abuse (NIDA) has identified a persistent research-to-practice gap in the implementation of evidence based prevention and treatment programs for substance use disorder [8]. For example, less than half of specialty programs that treat opioid use disorder (OUD) provide access to OUD medications, for which evidence soundly supports the effectiveness and cost effectiveness [6, 9]. Economic evidence, in particular, has become increasingly important in today’s rapidly evolving healthcare system. Greater availability of health insurance, combined with state and national parity requirements for insurance coverage of behavioral health conditions, plus new organizational delivery models such as integrated health care systems, all create opportunities for improved access to evidence based services. However, wide gaps remain in the implementation of such evidence-based practices. Given that new delivery, coverage, and payment models are increasingly important to how substance use disorder care is distributed and provided, little is known about how evidence – and, particularly, economic evidence such as cost effectiveness research -- could help close these implementation gaps and improve the application of evidence-based care.

Previous research has described specific barriers and facilitators to the use of research in health care delivery and policy. Much of these empirical studies have used interviews and surveys with governmental officials as well as other decision makers. Caplan and others identified two separate communities of researchers and policymakers that often have different timelines, incentives and needs [10, 11]. In a synthesis of policy knowledge translation research, Mitton and colleagues identified barriers such as misaligned incentives between researchers and policy makers, poor communication, and the asynchronous timing of research and policy-making [12]. The extent to which these previously characterized concepts apply to behavioral health and SUD research has not been described [13]. Moreover, effective ways to reduce the barriers to research use in policy (including resource allocation) may be unique for substance use disorder compared to other health domains that bear less stigma [14–16]. A recent multiple case study of opioid policy regarding research policy translation initiatives uncovered strategic themes focusing on building consensus around policy topics, long term engagement, and use of knowledge brokers [17]. This investigation, as well as other explorations of evidence based policymaking in mental health have not, in large part, focused on the impact of economic research [18]. These questions are particularly important at a time marked by rapidly shifting health care systems and a sense of urgency about the opioid epidemic, creating a potential “window” [19] of opportunity for developing and implementing evidence-based SUD policies.

### Investigation aims

The purpose of this study was to identify barriers and facilitators, attitudes, beliefs, and experiences surrounding the use of research related to the treatment and economics of substance use disorder. We explore concepts and perspectives that have not previously been formally studied, and highlight ways that researchers could bridge these gaps and optimize the design, translation, and dissemination of useful research.

## Methods

### Study design and sample

We conducted open-ended, semi-structured interviews with a purposive sample of policy makers. Policy makers were broadly defined as leaders and decision makers from a variety of sectors who might use research to influence healthcare related to SUD. We recruited informants using snowball sampling, starting with members of the advisory committee for the Dissemination and Policy Core of a Center of Excellence focused on the health economics of substance use disorder, HIV and Hepatitis C treatments funded by NIDA [20]. The committee was initially created to provide the Center with diverse perspectives on key policy and economic questions relevant to research. Each committee member, in addition to being interviewed, was asked to provide the names of other decision makers/informants. The final sample included decision makers from health care delivery systems, the insurance industry, the pharmaceutical industry, clinical care settings, federal and state governments, and patient advocacy organizations. The Institutional Review Board at the University of Pennsylvania approved the study. We used the Consolidated Criteria for Reporting Qualitative Research (COREQ) to guide collection, analysis and reporting of the data [21].

### Data collection

Interviews were conducted individually with each respondent in person, by video teleconference, or by telephone and ranged from approximately 45 min to 90 min, following a standard interview guide. The guide was designed by the investigative team with training in qualitative interviewing, health policy, and economics. Modified grounded theory, starting from the constructs of knowledge translation outlined by Lavis [22], was used to guide the development of the interview guide (see Additional file 1). Two investigators (ZFM and JM) piloted the interview guide with three local policy makers. These respondents were interviewed for the purpose of revising and refining the interview guide and were not included in the final sample. All subsequent interviews were conducted by one of the two investigators. The interviews addressed the use and usefulness of SUD research (and specifically probed on topics regarding SUD health economics research), the sources from which this research is accessed and reasons why it is used, barriers to and facilitators of use, and perceptions of the importance of translating this type of research into policy and practice. Each interview was audiotaped, transcribed, de-identified and entered into NVivo 11.0 (QSR, Doncaster, Australia) for data management and analysis. Participants were recruited until thematic saturation was reached, determined by consensus among study investigators during coding and analysis meetings. Eighteen interviews were conducted. As expected interview respondents were highly knowledgeable about the study topic and thematic saturation began to occur after the fifteenth interview.

### Analysis

We used modified grounded theory to analyze the transcripts [23]. This approach allowed for an analysis that was largely inductive, but was modified to allow for coding concepts associated with theories of research utilization, including those of Caplan and Lavis [10, 22], in policy making. First, members of the author team (ZFM, JM, NB, JW, CCC, EM) discussed eight transcripts and identified key concepts related to the use of research for policy. An initial codebook was created using these concepts as well as concepts determined a priori, including known barriers, facilitators and other characteristics associated with research use [12, 24, 25]. Two coders (EM and NB) piloted the coding. During the pilot process, additional concepts were identified and added to the coding scheme, and some nodes were collapsed or eliminated. All transcripts were double-coded to assess inter-rater agreement (92.99%). Disagreement was reconciled with input from and discussion among the entire analytic team.

Next, investigators created analytic memos for all nodes, describing key themes and a comprehensive list of relevant data points (quotations from interviewees). Using the constant comparison method, in which newly collected data were compared with categories that emerged from previously collected data, all memos were analyzed for consistency and disagreement. We examined differences in concepts for stakeholders by their employment type (government, health system, industry, clinical, and health insurance sectors). Findings were consistent rather than divergent across these categories, and so we report the findings at the level of the group as a whole. Last, all informants were invited (twelve attended) a conference held in June 2017, where they heard preliminary results and were given the opportunity to discuss findings with the investigators, which did not produce any new or contradictory perspectives.

## Results

Table 1 describes participants’ professional and demographic characteristics.

**Table 1:** Participant Characteristics (*N* = 18)

	N (%)
Sex	
Male	13 (72.2)
Female	5 (27.7)
Professional Background^a^	
Public (Government)	6 (26.1)
Private (Industry)	6 (26.1)
Nonprofit	11 (47.8)
Education	
PhD/MD	1 (5.6)
PhD	4 (22.2)
MD	6 (33.3)
JD	1 (5.6)
Master’s Degree	5 (27.8)
Other	1 (5.6)

^a^Some participants had multiple backgrounds

The analysis generated four themes that characterize the application of treatment and economic research in SUD healthcare policy and system decision making: 1) policy maker engagement with evidence and researchers; 2) strategic use and usefulness of research; 3) scientific rigor versus relevance; and 4) communication of evidence. Within each of these themes, the participants identified barriers, facilitators, current practice, and gave their perspectives on “ideal conditions” for research design, conduct and communication to produce policy decisions that are fully responsive to their policymaking needs. Table 2 summarizes these results and offers actionable steps.

**Table 2:** Key themes, representative quotations from policy makers, and actionable steps to generate policy-relevant research

Key Themes	Representative Quotations	Actionable Steps
Engagement with Evidence and Evidence Producers	*“I don’t hear about a particular research study until it’s published and I don’t have any opportunity to provide any kind of input into it. So, I think it’s kind of [important to shift] … to building more upfront engagement.”*	Involve policy makers, community members, and other stakeholders early in the research process. Cultivate informal networks including researchers, policy makers and other stakeholders.
Usability of Research	*“I always think cost effectiveness and return on investment is gonna’ be the thing that sort of turns the tide, and sometimes it is. But, what I’ve been surprised at is how powerful actually qualitative and anecdotal information is in policymaking. So, I think as important as cost effectiveness and ROI stuff is, it has to be accompanied with other kinds of evidence or frankly stories to humanize the issue.”*	Formulate research questions and study designs to answer the strategic goals of payers, health system leaders and providers, audiences that the study is intended to inform. Advance the use of qualitative research methods to promote the adoption of evidence-based policies for SUD.
Balancing Rigor and Relevance	*“For the decisions I need to make, it actually needs to be a bit less rigorous, because more rigor equals more time. I don’t need the exact answer out there to three decimal points. I need to be directionally correct.”*	Use study designs that prioritize timeliness to respond to the urgency of public health crises, such as the opioid use epidemic. With key informants, systematically test alternative phrasing for research terminology and jargon.
Communication of Evidence and Analysis	*“But the other thing I think researchers need to be ready to do is back it up, meaning testify if necessary. You never get academics to participate in any of the hearings or drafting of legislation. And I think if you’re a researcher and you care enough about this, you need to stop seeing yourself as so kind of above it all.”*	Promote researcher involvement in policy discussions. Communicate how research was conducted, how it applies to the policy maker’s organization or locality, and how it can be implemented. Within academic institutions, incorporate policy impact as an evaluation /promotion criteria.

### Theme 1: Engagement with evidence and evidence producers

Personal and professional relationships are key components of policy makers’ engagement with SUD research. Without prompting, 11 (61%) of participants noted that they reached out to their personal contacts and networks to identify research findings (in general) to use in policy making and practice. Many policymakers had created informal networks with researchers that were important, but required continuity and cultivation. When probed for their confidence in information that comes from researchers, some indicated that they trust only researchers with whom they have established relationships. Interviewees also explained that researchers must remain connected to policy makers to be ready to provide critical information when needed.

Other respondents (39%) noted that they attended conferences and webinars to learn about research. One interviewee mentioned a standing contract with a university as a way of accessing researchers and their findings. Specific to health economics as it related to SUD treatment research, 10 (56%) of interviewees identified “scanning the literature” or having someone else scan the literature for them. However, they expressed concern about accessing the appropriate literature to meet their specific needs. Repeatedly, they noted that “with so much out there”, they had trouble finding what they needed. Others said that they looked to summary reports such as those from the Congressional Budget Office (CBO) or the Centers for Disease Control and Prevention (CDC).

In addition to working through their informal personal and professional networks, interviewees had formal relationships with “in house” librarians or staff whose specific role was to interpret and synthesize data in a way that was responsive to the timely needs of the organization. Automated processes, such as subscription services as well as research briefs, published by trusted intermediary organizations such as the Centers for Disease Control, were sometimes used, but not always useful, largely because they are perceived as not addressing local needs (a key theme, discussed below).

### Theme 2: Usability of research

The respondents had differing viewpoints on the usability of economic research. Many of them acknowledged the importance of cost and economic data for any policy decision, be it at the organizational, state or federal level. These responses were often in direct response to interview questions that focused on the perceived importance of these types of data. One interviewee recounted her experience testifying before a legislature: *“They [legislature] do care about the cost offsets. They care about the cost benefits. They care about the return on investment in particular because they’re…at the state level [asking] ‘what return do we get for this?’ Particularly in substance abuse”.*

Decision makers repeatedly noted that the value of economic and SUD treatment research was defined by *how* it could be used to further a specific goal of the stakeholder. This strategic application of the research — either to answer a local question or to make a policy case to others – was identified as the driving force behind research use. These responses tended to arise organically during the interviews and were not direct responses to specific queries or probes. In many cases, the strategic motivation behind the use of research characterized the type of research information needed. For example, the budgetary needs of decision maker’s organization dictated the types of economic data needed. One health system leader stated that he needed to know the general “direction of the evidence”, more than the nuanced details, largely due to the planned strategic use of those data. *“[For my organization] evidence will be used as a blunt instrument: should I go left or should I go right?,”* he stated.

The delineation of population subgroups, particularly in the realm of mental health and substance use, was raised multiple times by stakeholders as an important way to bring data into a more usable frame for policy. As one respondent noted,

*“It doesn’t matter if you do CBT [cognitive behavioral therapy] or basket weaving – you’ve got to have the necessary conditions first. Then once you have that, then you step down to the next specialization. Is this a pregnant woman population? Is this criminal justice population? Is this an adolescent? You don’t necessarily change your entire policy based on one article that came out yesterday – there’s a thousand articles that come out every day.”*

A qualification expressed throughout these interviews, especially in reference to health economics research, was related to the need for decision makers to differentiate between generalizable information and local data. They commented on the limited usefulness of research data that might be generalizable to large populations, but not necessarily relevant to the specific needs of their own organization. One respondent noted the usefulness of “macro information that any state could use” as long as decision makers could “plug in their own numbers” during economic conversations regarding program decisions. Respondents across professional sectors noted that cost effectiveness studies rarely differentiate between social costs and organizational costs. As one noted, “So let’s be careful about the term cost effectiveness because it sounds like you’re talking about *our* cost.”

### Theme 3: Balancing rigor and relevance

Policy stakeholders spoke extensively about their frustrations with research that suffered from jargon, overly technical methodological approaches, poor timing (slow speed from idea to publication), and research designs or data sources that didn’t feel relevant to their needs. When discussing economic research in particular, respondents were frustrated by terminology such as quality adjusted life years (QALYs) used in cost-effectiveness research. One interviewee stated, “*I’ll tell you, one of things that is a problem for economic research– it’s basically analytic problem– you have things like QALYs– quality adjusted life years– and frankly, [expletive] like that. There’s nobody who’s interested in that, nobody*.” Another interviewee echoed this sentiment: ‘*First off, researchers need to use the vocabulary of policy folk and not as if they’re talking to each other. Very few people understand the term Q-A-L-Y. And – or worse, they abuse it.*’

Other decision makers, especially those who led organizations, reinforced this perspective, in which methodological rigor was viewed as potentially less important for policy as other factors. Interviewees alluded to the concept of “clean” research, which is useful “for the purposes of a grant,” compared to “messy” research, which may have more real-world applications. As an example, one stakeholder noted that perceived rigor of the research sometimes interfered with his organization’s needs, especially if rigorous research led to results that emerged after he needed them.

### Theme 4: Communication of evidence and analysis

With few exceptions, interviewees expressed an overall desire for researchers to be directly engaged in policy-making dialogues. They lamented the relative isolation of academics who may be unwilling to get involved in policy discourse. Stakeholders observed that academics often complete their peer-reviewed publications and see their contributions as stopping there. This sentiment is exemplified here: “*but the other thing I think researchers need to be ready to do is back it up, meaning testify if necessary. You never get academics to participate in any of the hearings or drafting of legislation. And I think if you’re a researcher and you care enough about this, you need to stop seeing yourself as so kind of above it all.”*

Specific to SUD research, stakeholders described researchers as needing to have “skin in the game” where investigators have specific professional and personal stakes in evidence-based policy. As one stakeholder stated: “*I really can’t stress enough that if you are doing research in these areas - which I hope somebody is - on prevention and messaging and successfully quitting [substance use] – what does that look like? I mean, you just have to be getting that information out, right? It’s not enough to just get it in a journal or something like that.”* This was a repeated theme, in which respondents noted the urgency for evidence-based solutions for SUD treatments and expressed frustration that researchers were relatively isolated from the policy process, which undermined progress toward effective policies.

Respondents recognized that researchers don’t always possess the tools or confidence to directly communicate results in policy forums, but sensed that many researchers would be willing to do so if provided the right opportunity, and some coaching and framing guidance. One medical provider and leader of SUD advocacy organization stated, “*What I’m finding is that there are a lot of academics who are … very civically engaged and want to use sort of the tools and skills that they have to advance particular kinds of policy change.*” This research user continued, “*I will say what I found is this kind of bimodal distribution of academics that are willing to play along with us. And it is people at the beginning of their career that are kind of very enthusiastic and maybe naively idealistic, but have a lot of energy and passion. And then it’s people late in their career who have tenure and maybe have more time, but also don’t feel that they’re putting themselves at risk by participating with an advocacy organization*.”

Policy makers suggested partnering with researchers in the effort to translate and communicate research. One state policy maker noted,

“*We had a policy campaign [in our state] to expand the medical marijuana program and we wanted to include severe chronic pain as one of the conditions to qualify because it’s a very narrow program right now. And we knew that the state legislature wasn’t really interested in fixing the medical marijuana program, but they were very concerned about opioid overdose deaths. And so, there’s a researcher who had done a study that states with medical marijuana programs had significantly lower rates of opioid overdoses. So, I reached out to that researcher and said, do you want to try to help us? And he did, but the work with him was, he wanted to talk about medical marijuana as pain management. And so, what I saw my job was saying, listen, the current policy moment in [the state capital] right now is about opioid overdoses and so that’s the frame we need to use, which he understood and was willing to do because it was consistent with his research*.”

## Discussion

We identified a range of perspectives from health policy makers and decision makers regarding the use and usefulness of SUD treatment and economic research. This work is timely in light of the opioid epidemic which has opened a window for the development and implementation of new policy approaches to SUD treatment and prevention. Ideally, the delivery of treatment would be guided by clinical and cost effectiveness evidence that could improve care for people affected by SUD. Health system leaders, public policy makers, providers, and insurers might use this evidence to implement best practices. We sought their perspectives on the use of such research. These insights and narratives coalesced around a few key themes, including researchers’ engagement with policy stakeholders, the strategic use of research, tensions between scholarship methods and local relevance, and communication of research by investigators.

The use of research in policy decision making has been explored across a variety sectors and topics [12, 25, 26]. However, prior research has emphasized the ways in which policy makers should better capitalize on existing research. Less fully documented are the ways in which research could more effectively attend to the needs and concerns of decision makers. In this paper, we shift the onus of action to researchers, suggesting ways that the academic community can engage in specific steps to help close the gaps between clinical and cost effectiveness research and care for patients with SUD. The themes we identified are consistent with both the model of strategic science proposed by Brownell and Roberto and the model of integrated knowledge translation described by Gagliardi [27, 28]. In the strategic science framework, the importance of relationships between researchers and policy makers is highlighted, starting with researchers developing questions that meets decision makers’ needs [28]. Gagliardi outlines collaboration between researchers and decision makers, and recommends the identification of partners with pre-established links to ease or expedite the interaction, the establishment of clear role expectations, the development of mechanisms that initiate and support dialogue, and joint efforts to assess progress and implement changes [15]. In this study, our informants were policy “stakeholders” rather than researchers, and these participants echoed the need for early and collaborative researcher-stakeholder cooperation which is also supported by NIDA’s strategic plan [8].

Participants identified a wish-list for engaging SUD policy research and researchers that also aligns with other frameworks for research use. They want research that incorporates useful, meaningful and clear metrics, as well as tools and to help them persuade peers and convey the importance and feasibility of their preferred policy solutions. They noted that if a policy was analyzed and appeared to be effective *and* cost effective, they wanted a locally-relevant model specifying how to implement policies that improve substance use disorder outcomes. They also placed a higher priority on rigor over timeliness, and expressed frustration with cost-effectiveness terminology that was not considered relevant. In many ways, this expressed need can be situated within the concept of the *problem-solving* mode of research utilization as defined by Weiss, in which evidence is used instrumentally to solve specific problems [29]. At the same time because of the limited relevance of much research to local needs [30], they stated that they often use research for broader conceptual purposes, which would align with Weiss’ *enlightenment* mode of research use [29]. An example, cited by multiple respondents, would be combining data with stories to reduce stigma that impedes progress in applying evidence based solutions to the treatment of patients afflicted by SUD, resulting in a minority of programs providing access to OUD medications despite the increasing availability of insurance coverage.

This study has some limitations. Although we identified themes common to participants, there may be limits to generalizability because of the snowball sampling strategy employed, particularly since policy makers with a stated interest in SUD were enrolled. The goal of this qualitative work, however, was not to understand the prevalence of specific perspectives, but to elicit the breadth and range of viewpoints. Participants may have been susceptible to social desirability bias in which they reported favorable perspectives on research because they were speaking to researchers. However, because most of the participants included critical perspectives on the use of research in their responses, we do not believe this limitation inhibited them from speaking candidly. The respondents were more educated than the average elected official. Although infectious disease (HIV and Hepatitis C) screening and treatment was a pre-specified topic for investigation, the analysis did not uncover specific ideas or concepts related to this topic that were distinct from the overall themes which emerged during the general discussion of substance use and economics evidence. Finally, an important limitation is that awareness, use, and attitudes toward evidence-based policies in SUD are likely evolve over time. Perceptions may change with publication of new evidence, media attention, passage of legislation, dissemination of resources, evolution of the current opioid crisis, or new SUD related public health challenges.

## Conclusions

In summary, policymakers want research and researchers to help them understand and be able to convey the importance and feasibility of various SUD policy solutions. For them, research must begin with engagement between researchers and end users, be visibly useful, be locally relevant; and be communicated via established relationships. Recommendations for investigators to achieve these goals include the following actionable steps: 1) partner with policy makers early in the research process, 2) formulate and use research designs to meet the strategic goals of end-users; 3) systematically test alternative phrasing of scientific terminology – particularly in the realm of cost effectiveness research – that allow end users to better understand and repurpose the data; 4) incorporate qualitative research methods to uncover the narratives that explain the context and relevance of evidence; 5) incorporate study designs that prioritize timeliness of results; and 6) promote and reward researcher involvement in policy discussions. Putting these opportunities into action will require the interest and effort of researchers as well as incentive structures that support them in professional and academic institutions [31]. We must continue to research how to best prioritize and implement the strategies that will close the gaps between SUD research and its use.

## Additional file

Additional file 1: Semi-structured interview guide and script. (PDF 194 kb)

## Abbreviations

CHERISH: Center for Health Economics of Treatment Interventions for Substance Use Disorder HCV and HIV
NIDA: National Institute on Drug Abuse
OUD: Opioid Use Disorder
QALY: Quality Adjusted Life Years
SUD: Substance Use Disorder
