# Supplementary material for: Strengthening partnerships between substance use researchers and policy makers to take advantage of a window of opportunity
Source: Subst Abuse Treat Prev Policy. 2019 Mar 4;14:12. doi: 10.1186/s13011-019-0199-0 (PMC6399875; doi:10.1186/s13011-019-0199-0)
Supplement: Supplementary file 1 — Semi-structured interview guide and script. (PDF 194 kb) [file 13011_2019_199_MOESM1_ESM.pdf]

Assessing policy stakeholder needs for economic research on substance use disorder

Interview Guide and Script

Thank you for agreeing to participate in this research study. The purpose of this phase of the study is to assess the needs of policymakers for economic research when making decisions about coverage for treatment of substance use disorder.

Before we begin, do you have any further questions regarding the study?

- When you are making decisions related to funding or care delivery of treatment of substance use disorder, what resources or tools do you use?
    - *Follow-up questions, if necessary:*
    - What other resources or tools would help you make these decisions?
    - What resources do you use when making decisions about Hepatitis C treatment?
    - What resources do you use when making decisions about HIV treatment?
- When thinking about new interventions, treatments, or practice patterns what evidence is helpful?

- Where do you seek data or information related to these decisions?

- What keeps you from accessing the data that you need to make these decisions?

- What are specific types of evidence do you need to make these decisions? Can you give an example?

- What are some ways that researchers might design their studies to be more helpful to policy makers like you who are making these decisions?

- Describe the communication that you have with researchers. Does it work? How could it be improved?

- In the era of the Affordable Care Act, have the factors affecting coverage decisions changed for your organization?
    - *Follow-up questions, if necessary:*
    - If so, what has changed?
    - Are there new research needs in the setting of the changing landscape?

- What is most striking, interesting, or bothersome about this topic to you?

- Do you have an adequate sense of the value of new interventions? Or cost implications? Is cost-effectiveness evidence useful?

- Can you think of a more useful representation of tradeoffs between costs and quality of treatments in your setting?

- What are the most significant issues related to the financing, organization, and delivery of care for SUD/HIV/HCV populations that you expect your organization to face three years from now? Five years from now?

- Do you see a role for researchers in helping address these issues? If so, how?

- How can the research community be most helpful in providing relevant, research-based evidence to you and your organization?

- Is there specific information related to SUD/HCV/HIV treatment costs and value not obtainable by policymakers (for either current or future anticipated needs)? Do you have suggestions for how to fill those gaps?

Identification and Implementation of Innovations

- To what extent does your organization try to identify evidence-based innovations in /HCV/HIV treatments?

- If so, how do you identify these innovations? To what extent do you rely on published peer-reviewed literature? Summaries of research and specific innovations in trade publications and other non-peer reviewed sources? Conferences and professional associations? Word of mouth?

- What information would your organization need in order to introduce a particular innovation?

- How do you evaluate the appropriateness of the innovation for your organization?

Sources of Information

- Are there key sources you rely on for research or other evidence on the cost and value of SUD/HCV/HIV treatments?

- In general, who/what do you consider trusted sources of technical information in terms of SUD/HCV/HIV? Are there common sources you consider untrustworthy in some way? Why?

- What do you think is the most useful format for receiving information about the results of a research study?

- Do you currently work with researchers (off-site or embedded)?

- If yes, how? How has that experience gone?
- What are the benefits and challenges of such collaborations? What makes for a good academic collaboration? Bad collaboration?
- What advice would you give to researchers and funders seeking to enhance the policy relevance of the research they undertake or support?
- What didn’t we ask that we should have?
